# A commentary on ‘A new designed full process coverage robot-assisted total hip arthroplasty: a multicentre randomized clinical trial’

**DOI:** 10.1097/JS9.0000000000001572

**Published:** 2024-05-08

**Authors:** Shu Li, Yong-Gang Bao, Bin Wu

**Affiliations:** a Department of Clinical Medicine, Jining Medical University; b Department of Orthopedics, Affiliated Hospital of Jining Medical University, Jining City, People’s Republic of China

*Dear Editor,*

I am writing in response to the article titled ‘A new designed full process coverage robot-assisted total hip arthroplasty: a multicentre randomized clinical trial,’ published in *International Journal of Surgery* ^1^. First, I would like to commend the authors and the journal for presenting an innovative study that significantly contributes to the field of orthopedic surgery.

The authors have meticulously detailed a novel robotic-assisted total hip arthroplasty (RA-THA) system, comparing its performance against manual total hip arthroplasty (MTHA)^2^. This multicenter study is robust, leveraging randomized clinical trials to enhance the credibility of its findings. The introduction of the TRex-RS system, utilizing preoperative computed tomography for precise prosthesis placement, is a commendable advancement. It addresses key challenges in hip arthroplasty, such as leg length discrepancy (LLD) and femoral prosthesis angulation, presenting a solution that potentially increases the accuracy of surgical interventions^3^.

However, I would like to point out some areas that could benefit from further discussion or improvement. The study notes no significant differences in various parameters, such as the Harris hip score and VAS (visual analogue scale) score post-operation between the RA-THA and MTHA groups. While the robotic system appears to excel in reducing LLD and femoral angulation, the clinical significance of these improvements remains slightly underexplored. The prolonged operative time in the RA-THA group also warrants a discussion regarding cost-effectiveness and the practicality of adopting such technology widely, considering the constraints of surgical time in many healthcare settings.

Another aspect that could be enriched is the discussion on the learning curve associated with the new robotic system. As RA-THA involves novel technologies and techniques, understanding the training required to achieve proficiency would be invaluable for institutions considering its adoption^4^.

Additionally, while the study beautifully outlines the technical successes of the robotic system, a deeper exploration into patient-centered outcomes would greatly enhance the article. Understanding how these technical improvements translate into patient satisfaction and long-term quality of life could provide a more rounded view of the system’s benefits.

In conclusion, the study presents a significant technological advance in the field of orthopedic surgery, offering a promising avenue for improving surgical accuracy and patient outcomes in hip arthroplasty. I thank the authors and the journal for their contributions to expanding the horizons of medical technology and look forward to seeing further research on this topic, particularly in terms of long-term outcomes and broader applicability.

## Ethics approval

Not applicable.

## Consent

This manuscript is a comment. Don’t need patient consent.

## Sources of funding

This work was supported by the Cultivation Plan of a high-level scientific research project of Jining Medical University [grant number JYGC2021FKJ016], the Jining Key Research and Development Program Fund [grant number 2021YXNS029], Jining Key Research and Development Program Fund [grant number 2022YXNS129], and the Special Clinical Research Program of the attending physician team at the Affiliated Hospital of Jining Medical University [grant number ZZTD-MS-2023-04].

## Author contribution

S.L. and Y.-G.B.: writing of the manuscript; B.W.: concept and design.

## Conflicts of interest disclosure

The authors declare no conflicts of interest.

## Research registration unique identifying number (UIN)

Not applicable.

## Guarantor

Bin Wu.

## Data availability statement

The correspondence is based exclusively on resources that are publicly available on the internet and duly cited in the ‘References’ section. No primary data were generated and reported in this manuscript. Therefore, the data have not become available to any academic repository.

## Provenance and peer review

The paper was not invited.
